# Complex single step skull reconstruction in Gorham’s disease - a technical report and review of the literature

**DOI:** 10.1186/s12893-015-0014-4

**Published:** 2015-03-11

**Authors:** Victoria Ohla, Ahmed B Bayoumi, Markus Hefty, Matthew Anderson, Ekkehard M Kasper

**Affiliations:** 1 Department of Neurochirurgie, Universitätsklinikum Essen, Hufelandstraße 55, 45147 Essen, Germany; 2 Division of Neurosurgery, Beth Israel Deaconess Medical Center, Harvard Medical School, 110 Francis Street, LMOB Suite 3B, Boston, MA 02215 USA; 3 Department of Pathology, Beth Israel Deaconess Medical Center, Harvard Medical School, Boston, MA USA

**Keywords:** Gorham’s disease, Osteolytic disorder, Cranioplasty

## Abstract

**Background:**

Gorham’s disease is a rare osteolytic disorder characterized by progressive resorption of bone and replacement of osseous matrix by a proliferative non-neoplastic vascular or lymphatic tissue. A standardized treatment protocol has not yet been defined due to the unpredictable natural history of the disease and variable clinical presentations. No single treatment has proven to be superior in arresting the course of the disease. Trials have included surgery, radiation and medical therapies using drugs such as calcium salts, vitamin D supplements and hormones. We report on our advantageous experience in the management of this osteolyic disorder in a case when it affected only the skull vault. A brief review of pertinent literature about Gorham’s disease with skull involvement is provided.

**Case presentation:**

A 25-year-old Caucasian male presented with a skull depression over the left fronto-temporal region. He noticed progressive enlargement of the skull defect associated with local pain and mild headache. Physical examination revealed a tender palpable depression of the fronto-temporal convexity. Conventional X-ray of the skull showed widespread loss of bone substance. Subsequent CT scans showed features of patchy erosions indicative of an underlying osteolysis. MRI also revealed marginal enhancement at the site of the defect. The patient was in need of a pathological diagnosis as well as complex reconstruction of the afflicted area. A density graded CT scan was done to determine the variable degrees of osteolysis and a custom made allograft was designed for cranioplasty preoperatively to allow for a single step excisional craniectomy with synchronous skull repair. Gorham’s disease was diagnosed based on histopathological examination. No neurological deficit or wound complications were reported postoperatively. Over a two-year follow up period, the patient had no evidence of local recurrence or other systemic involvement.

**Conclusions:**

A single step excisional craniectomy and cranioplasty can be an effective treatment for patients with Gorham’s disease affecting the skull vault only. Preoperative planning by a density graded CT aids to design a synthetic bone flap and is beneficial in skull reconstruction. Systemic involvement is variable in this patient’s population.

## Background

Gorham’s disease is a rare and potentially disabling osteolytic disorder. It is characterized by uncontrolled proliferation of non-neoplastic vascular or lymphatic tissues, leading to progressive resorption and replacement of osseous matrix which may extend to the adjacent tissues [1,2]. It was first described by Jackson in 1838 who reported a case of “boneless arm” and much later presented as a distinct clinical syndrome by Gorham and Stout in 1954, who characterized its main pathological features [3,4]. Other terms such as “massive osteolysis”, “disappearing bone disease” and “phantom bone disease” have also been used [5,6]. Gorham’s disease usually occurs in children and young adult patients, most commonly in the 2nd and 3rd decades [7-9] although any age group may be affected (with cases reported spanning from 1.5 to 72 years) [8] and without any race or gender predilection [2,10].

Histologically, Gorham’s disease is characterized by inflammatory osteolysis of bony segments which are then replaced by localized proliferative lymphatic channels [11]. These osteolytic characteristics may be accompanied by several types of non-neoplastic developmental vascular malformations, including capillary, venous and lymphatic malformations without endothelial cell proliferation [10,12]. Here, we present a case of Gorham’s disease affecting only the skull vault which was managed by a single-stage craniectomy and skull reconstruction, using a synthetic bone flap generated preoperatively via distinct computer aided planning technique. To put this into an appropriate context we included a review of the pertinent literature.

## Case presentation

**Figure 1:**
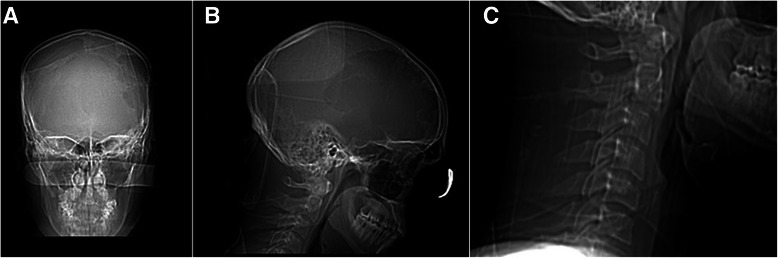
**Plain skull films as obtained from CT-scouts: (A) Anteroposterior and (B) lateral projections show the skull preoperatively together with a view C) of the upper cervical spine (lateral view).** No other lesions were evident, nor did we observe cervical fractures or misalignment. Sagittal and coronal sutures are visible.

**Figure 2:**
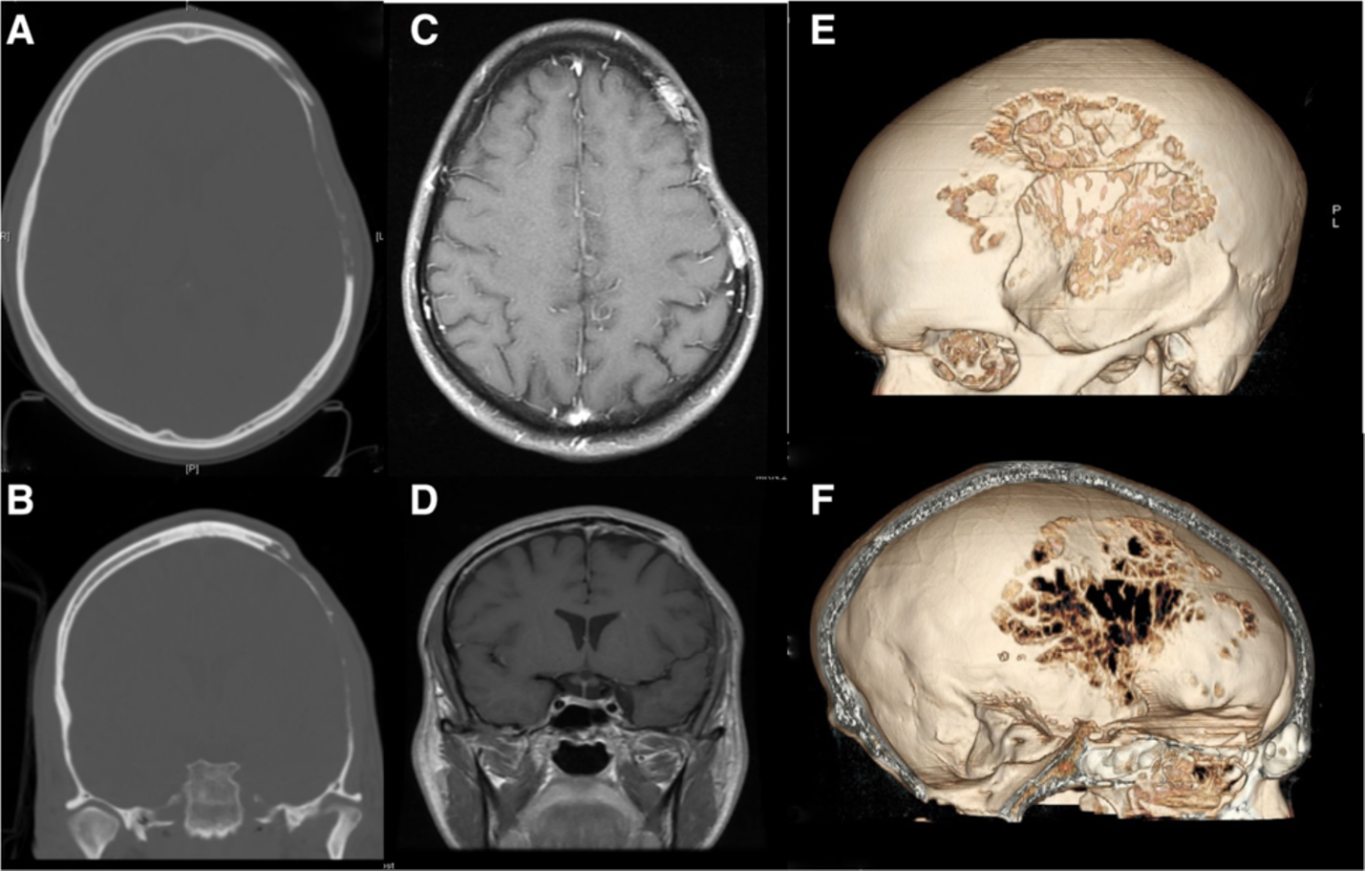
**Preoperative images. A**. and **B**. Preoperative CT of the skull bone window axial and coronal respectively showing frontparietotemporal bone thinning, erosion and defect. **C**. and **D**. Preoperative MRI brain T1-WI with contrast showing the marginal enhancement. **E**. and **F**. CT skull with 3D reconstruction showing the variable degrees of osteolysis from outside and inside respectively.

**Figure 3:**
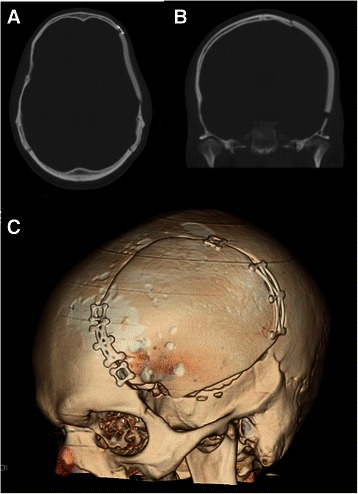
**Postoperative CT skull images following excision and reconstruction. A)** axial view of bone window, **B)** coronal view of bone window and **C)** CT skull with 3D reconstruction.

A 25-year-old Caucasian man presented with a skull defect over the left frontotemporal region that had progressively enlarged over a 3-year period. He complained of mild dull local aching headaches with short term memory impairment of one year duration, occasional visual symptoms, and subjectively decreased hearing. Symptoms were explained by the polysubstance abuse and the patient’s past medical history of depression. General physical examination was unremarkable with the exception of a clearly visible osseous depression of the left frontoparietotemporal region associated with mild tenderness on palpation and interspersed softness embedded in a thinned but firm skull. The patient was neurologically intact. X-ray of the skull (Figure 1A & B) showed widespread loss of calvarial structure secondary to an osteolytic process. Subsequent head CT scan (Figure 2A, B & F) showed the typical features of a patchy erosive osteolysis of the left frontoparietotemptoral skull region indicative of an active underlying process. Coronal, sagittal and lambdoid sutures were shown to be patent radiographically in skull-scouts (Figure 1A & B) and CT-cuts (Figure 3A & B) in order to exclude craniosynostosis. Post-contrast MRI study revealed marginal enhancement of the calvarial defect with no evidence of brain invasion or soft tissue component. (Figure 2C & D) Bone survey as well as chest x-ray were done to exclude skeletal and pulmonary involvement, respectively. Furthermore, the metabolic and chemical labs’ profile of the patient did not reveal any abnormality indicative of metabolic or endocrinologic disease. This study was approved by the institutional review board (IRB) of our hospital using an IRB protocol number (2013-P-000253/3). An informed patient’s consent was obtained to submit this article to the journal in order to be published.

**Figure 4:**
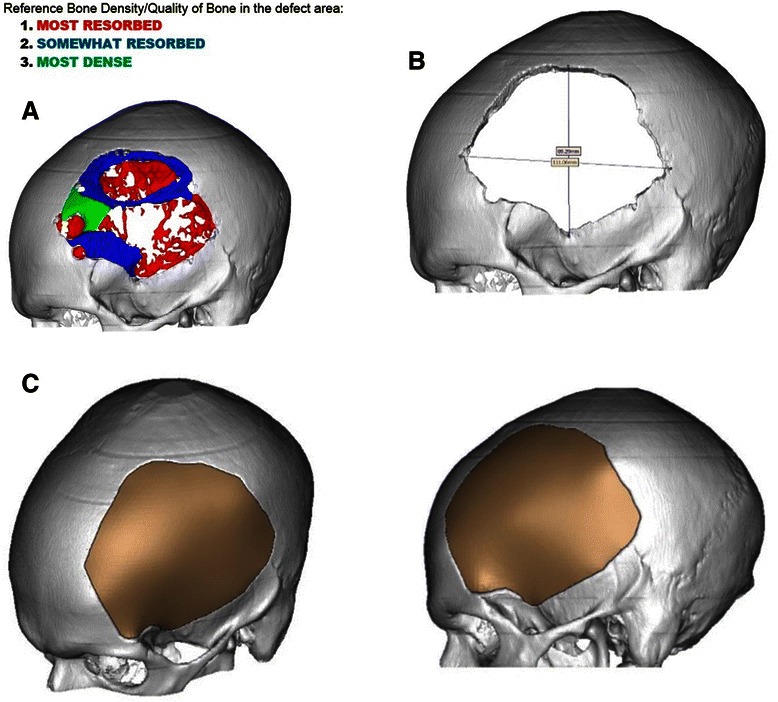
**Preoperative planning. A**. Variable densities of the resorbed bone are illustrated in different colors based upon CT. **B**. The measurements of the expected bone defect after removal of all regions (1, 2 and 3) giving an area of 90 × 110 mm which is the size of the designed implant synthetic bone flap. **C**. The expected final design of the bone flap replacing the defect and preserving the same skull contour.

Based on a density graded CT scan, the severity of erosion at the affected skull region was determined. A custom made cranioplasty allograft was designed preoperatively (Figure 4) using a digital computerized software (Stryker®, Kalamazoo, Michigan) to define the size, site and shape of the synthetic bone flap enabling a single-staged surgery of excisional craniectomy, allograft duraplasty and synchronous skull reconstruction. (Figure 3) The pre-fabricated implant was made of Poly Methyl Methacrylate (PMMA). This patient’s pre-manufactured implant configuration was designed using specific software (Mimics, Materialise Company, Belgium) which generated allograft models based on variable degrees of osteolysis seen in the affected part of the skull bone on CT. The extent of calvarial bone to finally be excised and replaced was decided from a density-graded CT scan. To this end, we randomly selected 10 representative points within diseased and healthy skull regions, and obtained the respective Hounsfield units. This yielded a range of bone density values. The mean value for diseased bone was 342 HU (range: 158 to 643 Hounsfield units) whereas the mean value for healthy bone was 1489 HU (range: 1274 to 1630 units), respectively.

To generate the final allograft construct, we then color-coded the prospective craniectomy area on the CT scan using the highest obtained density value of diseased skull (643 Hounsfield units). We used this particular value as a cut off since its density was about half that of the mean value obtained from the density measurements of healthy bone. A visual overlay was then used to confirm that the chosen implant shape matched all areas of diseased bone.

**Figure 5:**
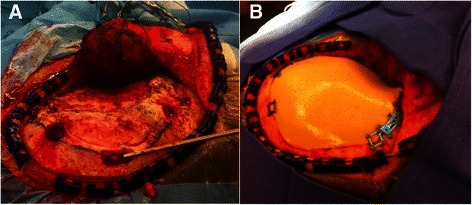
**Intraoperative images. A**. Exposure of the bony lesion following temporalis muscle separation. **B**. Final view following surgical excision and reconstruction using a synthetic bone flap fixed to the surrounding apparently healthy bone with miniplates and screws.

Surgery as such was then performed in a standard fashion. The patient underwent general endotracheal anesthesia and was position supine in three point skeletal fixation pins (Mayfield). Preoeprative density graded CT and MRI data were loaded on an intraoperative image guidance system (BrainLab) to map the affected area onto the scalp. The incision was planned accordingly and a conventional myocutaneous flap was raised. The affected area was visually identified. The optimal extent of the resection was taken from the CT and sketched onto the bony surface. For corroboration, the pre-generated bone allograft was put as a stencil onto the marked and affected skull area and it was confirmed that it could be used for the ensuing craniectomy. This way we could excise a bone segment that matched precisely the custom made implant for later repair. After standard craniectomy employing a side cutting saw (Anspach, DePuy; West Chester, PA), we separated the affected bone from the underlying layer of dura and repaired a very small dural defect with a pericranial autograft before we proceeded immediately with vault reconstruction using the prefabricated bony allograft (see Figure 5). The wound was hemostased and closed in layers without the need of subgaleal drains. The patient recovered from anesthesia immediately in the operative room. The postoperative period was uneventful and the patient showed no neurological deficit.

### Pathology

**Figure 6:**
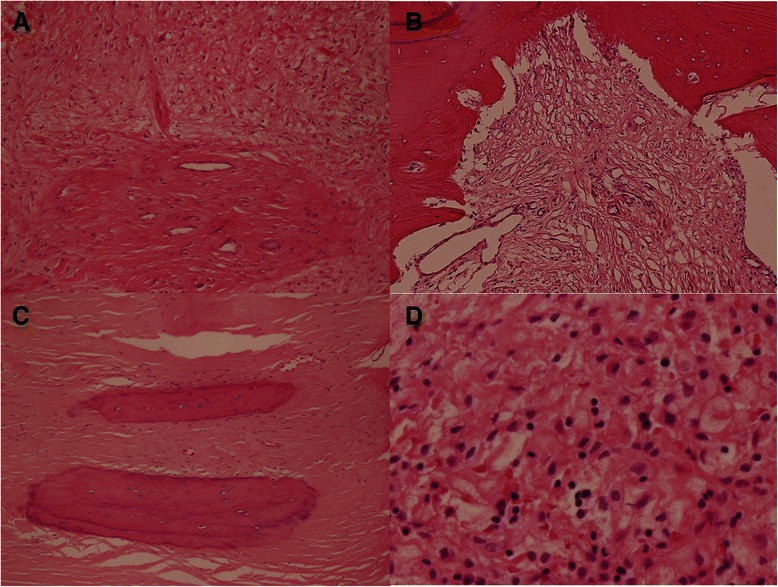
**Histopathology. A)** Replacement of bone by fibrous tissue with variable amount of collagen deposition and vascularity. (H&E, 10X objective) **B)** Presence of marked number of thin-walled vessels next to an area of active bone resorption. The blood vessels are predominantly capillaries, but smaller arterioles and venules are also seen. (H&E, 10X objective) **C)** Bone replacement by dense fibrous tissue with occasional small blood vessels. (H&E, 10X objective) **D)** Patchy area of chronic inflammatory infiltration with scattered foamy macrophages. (H&E, 40X objective).

Routine hematoxylin and eosin was performed on formalin-fixed, paraffin-embedded sections after decalcification of the submitted bone specimen. The sections revealed distinct areas of bone resorption with replacement by fibrous tissue with variable degrees of vascularity and collagen depositions (Figure 6A). In focal areas adjacent to zones of active bone resorption were numerous thin-walled, predominantly capillary-sized blood vessels (Figure 6B). In other areas, the zones of bone resorption were composed of densely collagenized fibrous tissue with interdispersed small blood vessels (Figure 6C). Beyond these characteristic features there were patchy areas with chronic inflammatory cells, including foamy macrophages (Figure 6D).

The postoperative period was uneventful without local wound complications or any neurological deficit and the patient was discharged four days postoperatively. Over a two-year follow up period, the patient did not show any evidence of resorption of the adjacent skull bone or any other skeletal involvement.

## Discussion

Gorham’s disease is a non-hereditary progressive osteolytic disorder that typically affects bones with subsequent lymphatic vascular malformation [13,14]. Gorham’s disease can be monostotic or polyostotic, however multicentric involvement is rare [15,16]. The most commonly involved sites are the mandible (15%), scapula (10%), ribs (12%), humerus (8%), pelvis (10%), femur (11%) [17] and less commonly the skull [18]. Clinical presentations vary based on the site of bone involvement and presence of systemic manifestations. To our knowledge, less than 30 cases of Gorham’s disease with any skull involvement, including this case report, have been reported in the literature.

Table 1 Published case reports of Gorham’s disease involving the skull [15,18-38].

**Table 1:** **Review of reported cases of cranial involvement in Gorham’s syndrome**

Reference	Age of onset	Gender	Location	Symptoms	Treatment	F/U outcome
Chiang et al. [19]	46	Male	Occipital bone	No neurol. Symptoms.	-	-
Wildförster et al. [21]	-	female	Squamofrontal	No neurol. symptoms		-
Zhang et al. [20]	40	Male	Parietooccipital region	No neurol. symptoms		Stable
Kawasaki et al. [22]	29	Female	Left temporal bone, facial mandibular and vertebral bones	Pain, hoarsenes, swallowing disturbace, postural instability of the neck and associated dyspnea and dysphagia, deafness and dumbness, left facial palsy, loss of vision	At age 34 years radiation therapy (total, 31 Gy) was performed. At age 35 years, further irradiation of the skull base (total, 28 Gy) was tried, but the osteolytic lesion expanded further. At the age of 36 years posterior cranio-vertebral fixation, tracheotomy and gastrostomy were performed because of postural instability of the neck and	Death
Chai et al. [23]	38	Male	Fronto-parietal	-	Cranioplasty	
Lo et al. [24]	23	Male	Left parietal region	No neurol. symptoms	Left parietal craniectomy was performed, Reconstructive surgery with artificial bone graft will be scheduled in the next hospital course, 3 months.	Stable
Papeix et al. [25]	40	Male	Parietal pone	No neurol. symptoms	Radiotherapy and surgery	-
Rao et al. [26]	20	Female	Left parietal bone	No neurol. symptoms	-	-
Frankel et al. [27]	14	Female	Calvarium	Rhinorrhoe, sensorineural hearing loss, immobile left palate, atrophy of the left side of the tongue with fasciculations	2340 cGy in 13 fractions	12 months, stabilisation and sclerosis
Hasegawa et al. [28]	49	Male	Left parietal bone	Headache	Surgery	-
Parihar et al. [29]	35	Female	Left parietal bone	No neurol. Symtoms	Left parietal craniotomy, cranioplasty	-
Iyer et al. [30]	58	Female	Frontal bone	Headache, vomiting, delirium, rhinorrhoea, meningitis	Pt. refused surgery	-
Girisha et al. [31]	16 months	Female	Calvaria	Developmental delay, failure to thrive	-	-
Kurczynski et al. [32]	14	Female	Left orbit, zygoma, mandible, sphenoid, and occiput	Left enophthalmia	Radiotherapy with 2000 rad to the entire skull, mandible, and upper cervical vertebrae	24 months, no further progression, slight remineralization
Khorsovi et al. [33]	62	Male	Occipital bone	No neurol. symptoms	Total of 4000cGy	24 months, arrest of disease process with new bone formation
Mawk et al. [18]	7	Male	Right skull base and cervical spine	Neck pain, lymphatic fluid within middle ear spaces and paranasal sinuses.	Surgery, 4140 cGy	3 months, no clinical or radiological progression
Plontke et al. [34]	54	Female	Skull Base	Right hearing loss	Cranio-cervical stabilisation, radiation total 30,6 Gy	8 months, no clinical or radiological progression
Girn et al. [15]	2	Female	Skull base	Clinical signs mimicking raised intracranial pres- sure and deafness	Halo application disease process did not respond to palmidronate and radiotherapy ( Five courses of radiotherapy with a dose of 35Gys in 20 fractions)	Continuous disease process, death
Schiel et al. [35]	14	Female	Posterior wall of the maxillary sinus, the orbit and base of the skull as fas as the apwx of the os petrosus	Right maxillary pain	Removal of right palatal mucoperiosteum and 40 Gy total	77 months, No evidence of further bone lysis
Hernández-Marqués et al. [36]	2	Male	Temporal bone	Secondary cerebrospinal fluid (CSF) leakage	Patient required two surgical interventions. The second intervention included mastectomy and placement of a patch and a lumbar drainage device during 50 days, after which the leakage ceased	-
Mowry et al. [37]	29	Female	Left temporal bone	Intermittent aural fullness, egophony, tinnitus bilaterally	-	-
Tsutsumi et al. [38]	82	Female	Bilateraly parietal regions	Painless scalp depressions	Open biopsy for histological verification	-

### Pathogenesis

The pathogenesis of Gorham’s disease remains poorly understood and a number of possible causes have been reported in literature. While Radhakrishnan and Rockson [6] suggested that Gorham’s disease is a disease of disordered lymphangiogenesis, Aviv and colleagues [39] suggested that it might occur independently from disseminated lymphangiomatosis, therefore representing two varieties of a rare disease etiology. Pathophysiological aspects regarding the presence or absence of osteoclasts in pathological tissue [40] as well as effects of hyperemia and changes in local pH-stimulating hydrolytic enyzmes remain controversial [41].

While Gorham and Stout [42] originally suggested that “osteoclastosis” was not a necessary feature, Foult and colleagues [43] pointed out that osteolysis occurred secondary to angiomatosis, and Spieth and colleagues [44] demonstrated a clear relationship between osteoclast activity and Gorham’s disease. This is further corroborated by the work of Möller and colleague [45], who described a large number of multinucleated osteoclasts with hyperactive resorptive function in his patients.

To determine the presence of blood and lymphatic vessel markers on the endothelial cells of the pathological proliferating vasculature in Gorham’s disease Hagendorn and coworkers [46] stained specimens for specific makers such as panendothelial marker CD 31 (platelet endothelial cell adhesion molecule), lymphatic vessel endothelial hyaluronan receptor-1 (LYVE-1), and VEGF receptor (VEGFR)-3. Over 90% of endothelial cells expressed CD-31 and were also found staining positive for LYVE-1, suggesting that the proliferating vasculature associated with Gorham’s lymphangiomatosis consists predominantly of lymphatic endothelium [46].

### Diagnosis

#### Clinical diagnosis

The reported clinical manifestations of Gorham’s disease were quite variable and largely depend on the site and extent of involvement. The presentation may be limited to local symptoms, such as pain and swelling of the affected extremity, soft tissue atrophy, and weakness of the involved limb or pathological fractures. However, systemic involvement, such as respiratory or neurological complications, was also frequently reported [7-9,16,44,47,48].

The neurological symptoms of Gorham’s disease vary greatly. Skull involvement may lead to progressive headache, migraines [5], nausea, vomiting, otitis media or recurrent episodes of meningitis secondary to chronic cerebrospinal fluid leakage [5,49]. Furthermore, some patients with temporal bone involvement may have auricular fullness, tinnitus, hearing loss or deafness [15,34,37]. Vertebral column involvement leading to pathological fractures, spine deformities and/or paraplegia has also been reported [7,24,50].

#### Radiologic diagnosis

Skull X-rays may initially show radiolucent foci which may subsequently extend into progressive dissolution and disappearance of a portion of the calvarial bone [24]. The osteolysis may extend to the contiguous bone and cross the intervening joint [24]. Kotecha and colleagues [51] emphasized the advantage of using quantitative computed tomography in the assessment of bones in patients with Gorham’s disease [52]. Among other benefits, it assesses the stage of the disease and aids in the decision-making processes at the time of initiating a particular treatment regimen also allowing to monitor individual patient response to any given therapy [53].

In addition to CT scans, thin cut fat-suppressed MRI T1-weighted contrast enhanced images help in visualizing a reticular pattern typical for the vascular component of the lesion [38].

Tc-99 scintigraphy is suitable in tracking the course of the disease activity as it may demonstrate increased uptake of radiopharmaceutical agents during initial active stages of the disease and subsequently show areas of decreased uptake corresponding to the diminished bone region in later stages of the disease or in response to treatment [7,16,44]. Torg and coworkers [12,54] classified Gorham’s disease progression by radiographic criteria allowing the differentiation of four sequential stages:

1. An initial stage in which radiolucent foci resembling patchy osteoporosis are present.
2. A second stage defined by an increase of bone deformity along with progressive loss of bone mass.
3. A third stage in which the cortical layer is disrupted by endothelial invasion into adjacent soft tissues or joints
4. Lastly a stage characterized by some shrinkage of the ends of affected bones.

#### Differential diagnosis and work up

The diagnosis is made via a combination of suspicious clinical and radiologic data as well as distinct histopathological features in conjunction with the exclusion of other hereditary, traumatic, metabolic, neoplastic, endocrinologic, infectious and inflammatory sources of osteolyses [55,56].

Although other osteolytic disorders of the skull (such as multiple myeloma, osteolytic metastases, juvenile Paget’s disease, eosinophilic granuloma and brown tumor) may show similar imaging findings, the CT, MRI and Tc-99 findings in combination with the long asymptomatic clinical course facilitate the differentiation of Gorham’s disease. Identifying areas of distinct vascular or lymphatic proliferation in early disease stages or the transformation to fibrous tissue in late disease stages can be achieved by generous biopsies of the affected bone and is essential for the unequivocal diagnosis of Gorham’s disease [42,57].

To this end, Heffez and colleagues [58] published a case report in which they suggested specific criteria distinguishing Gorham’s disease from other diseases of bony destruction which include:

- Positive biopsy with the presence of angiomatous tissue
- Absence of cellular atypia
- Minimal or no osteoblastic response or dystrophic calcifications
- Evidence of local bone progressive osseous resorption
- Non-expansile, non-ulcerative lesions
- Absence of visceral involvement
- Osteolytic radiographic pattern
- Negative hereditary, metabolic, neoplastic, immunologic, or infectious etiology.

The differential diagnosis should further include, but is not limited to: Paget’s disease, metastases, angiosarcoma, essential osteolysis and progressive parietal bone thinning. The latter is an age-related benign process not associated with metabolic or endocrine abnormalities and is usually seen on imaging as an incidental finding [59-61]. In contrast to Gorham’s disease, progressive thinning of the outer aspect of the vault is the main feature of biparietal thinning, occurring in pediatric skulls, although this has also been described in adults [62]. Differential diagnosis in children should include: juvenile fibrosarcoma, juvenile fibromatosis, and chondromyxoid fibroma [63] in Hajdu-Cheney-syndrome [64] which is a rare fibroblastic tumor with a predilection for the scalp of infants.

##### Management

Current treatments are only experimental as no single treatment has proven to be superiorly effective in arresting the course of the disease owing to its unpredictability [2]. Spontaneous arrest [65] or regeneration [66] of the destroyed bone without treatment has been reported [17,67,68], although the disease process generally requires multiple treatment attempts. [15] This may be particularly relevant in cases in which vital organs such as the spinal cord or lungs are involved, the latter of which can even result in pleural effusions or chylothorax [69]. However, the progressive involvement of vital structures in some cases may be fatal [2,70,71], resulting in an overall mortality of approximately 13.3% [72]. The prognosis of Gorham’s disease is otherwise considered to be good when disease is limited to the limbs or pelvic bones [73-76].

#### Surgical management

Surgical intervention has been suggested as the method of choice and includes resection of the lesion and possible re-grafting using various constructs [16,77-80]. However in the advanced stages of the disease, surgical procedures may be limited by technical issues such as the lack of bone substance for fixation of autologous or alloplastic material [5] or by the extent of systemic involvement. The pre-fabricated implant we used in our case allows a better cosmetic outcome by providing the exact natural skull contour compared to the conventional use of mesh and bone cement with excellent patient’s satisfaction. Although it may take more time preoperatively to design the compatible shape of the skull graft, it may save a lot of time intraopreratively to do both craniectomy and reconstruction in the same session applying the preformed skull implant precisely to replace the defect following the excision of the pathological bone. As there is no need for cement preparation and allograft molding this minimizes intraoperative time. The implant used in our case was formed of Poly Methyl Methacrylate (PMMA) which is known to have adequate impact resistance similar to native skull bone [81] with less risk of bone resorption compared to autologous bone flaps [82]. Furthermore, the pre-fabricated PMMA allows the surgeon to avoid any cement preparation phase, with its subsequent exothermic reaction which must be alleviated with cooling-irrigation to minimize the risk of thermal injury to the underlying structures such as the dura and/or the brain [83].

A limitation of this technique might be the high cost of such detailed preoperative planning when using density-graded CT scanning with 3D reconstruction as well as designing a patient-specific implant. Beyond this, its use is highly elective as the lag time makes it not suitable for neurosurgical emergencies (e.g. compound depressed skull fractures).

When planning surgery for patients with Gorham’s disease, certain precautions should be considered, as they may influence surgical management and strategies. Anesthesia induction must be done cautiously, as patients with maxillary or mandibular bone involvement may have difficult endotracheal intubation, which can be especially difficult in pediatric age groups. Protection of the spine is also important during induction and positioning [8]. Furthermore, postoperative ventilatory problems have been reported, emphazising that extubation has to be planned carefully and may involve prolonged intensive care management, as chylothorax is a possible life threatening complication that may occur even postoperatively.

Reconstruction techniques using prostheses seems to be effective despite potential obstacles since Woodward and colleagues [80], Kulenkampff and colleagues [73] and Paley and coworkers [84] have reported that the progression of adjacent disease has led to failure of reconstructions.

#### Conservative management

Based on the experience of Vinee and colleagues [16], medical treatment with hormones in combination with calcium salts and vitamins alone seem to be inefficient. Other treatment options include drug management and have been attempted using bisphosphonates, due to their antiosteoclastic and antiangiogenic activity. Lehmann and coworkers [85] reported a case of Gorham’s disease that was successfully treated with bisphosphonates for a period of 17 years. Hammer and colleagues [86] reported on bisphosphonate monotherapy (30 mg intravenous/3 months) controlling the disorder during a two year follow-up period. A successful conservative management was also reported by Avelar and colleagues [87], whose patient received monthly intravenous bisphosphonate infusions (at a dose of 4 mg) in addition to daily calcium (500mg) and vitamin D (400 UI) over the course of one year, showing maintenance of bone volume and symptomatic improvement of pain.

Interferon may also be useful because of its antiangiogenic effects [41] and its use has been reported by Dupond and colleagues [88] who treated a patient successfully based on a dosage of 7.5 to 15 million IU 3x/week over 5 years. However, this is contrary to results by Deveci and coworkers [89] who reported on a patient who died 4 months after the time of diagnosis, after being treated with interferon alpha-2b and bisphosphonates.

In the case we are presenting here, the patient did not need to receive any adjuvant radiotherapy or complementary medical treatment affecting bone remodeling, since disease was limited to one site only which was treated by excision. Girn and colleagues [15] reported on the management of a two-year-old girl with skull base and cervical spine involvement using radiotherapy and pamidronate therapy but this regimen resulted in failure to arrest the disease process and subsequent failure of surgery providing stabilization. In contrast, Heyd and colleagues [90], demonstrated that radiation therapy with the addition of intravenous zoledronic acid therapy may prevent the progression of the disease in 77% to 80% of cases with applied total doses ranging from 30 to 45 Gy. Similar results were presented in case reports by other authors (Bruch-Gerharz et al. [12], Johnstun et al. [91], Browne et al. [92] and Dunbar et al. [93]), who all came to the conclusion that radiation therapy in moderate doses (40-45 Gy at 1.8 Gy to 2 Gy per fraction) is effective. Due to the increased risk of radiation-induced secondary neoplasms and severe delayed toxicity, judicious use of radiation therapy is advised particularly in young adults and children [94,95].

## Conclusion

Gorham’s disease is one of the rare osteolytic disorders which may affect the skull or any other bone with or without systemic involvement. Surgical management by an excisional craniectomy and synchronous skull reconstruction is an effective and safe modality of treatment for Gorham’s disease presented with a solitary skull lesion. Preoperative planning by a density graded CT and special software to design a synthetic bone flap allows for single step reconstruction in this patient’s population for elective settings and complicated diseases such as Gorham’s, this seems to yield superior cosmetic results.
